# Supplementary material for: Breastfeeding Practices and Postpartum Weight Retention in an Asian Cohort
Source: Nutrients. 2024 Jul 8;16(13):2172. doi: 10.3390/nu16132172 (PMC11243595; doi:10.3390/nu16132172)
Supplement: Supplementary file 1 [file nutrients-16-02172-s001.zip › nutrients-3071962-supplementary.pdf]

Supplementary Table S1. Comparison of participant characteristics between those with complete and incomplete data at 6m and 12m pp

Variables	Complete data (n = 332)	Incomplete data (n = 216)	P value
PPWR at 6m, kg, mean ± SD	2.9 ± 5.2	3.5 ± 6.8	0.434
PPWR at 12m, kg, mean ± SD	2.6 ± 4.9	3.4 ± 6.3	0.271
Age at consent, year, mean ± SD	31.2 ± 3.6	30.4 ± 3.9	0.020
Pre-pregnancy BMI, kg/m^2, mean ± SD	22.5 ± 4.0	24.3 ± 5.3	< 0.001
Intervention group, n (%)			0.586
Control	106 (31.9%)	77 (35.8%)	
Facebook	115 (34.6%)	67 (31.2%)	
Midwives	111 (33.4%)	71 (33.0%)	
Ethnicity, n (%)			<0.001
Chinese	265 (79.8%)	111 (51.6%)	
Malay	30 (9.0%)	72 (33.5%)	
Indian	21 (6.3%)	23 (10.7%)	
Others*	16 (4.8%)	9 (4.2%)	
Education Level, n (%)			
Post-Secondary and below	63 (19.0%)	75 (34.9%)	<0.001
University and above	269 (81.0%)	140 (65.1%)	
Marital Status, n (%)			
Married	328 (98.8%)	204 (96.2%)	0.069
Single/Divorced	4 (1.2%)	8 (3.8%)	
Employment Status, n (%)			0.477
Employed	311 (93.7%)	198 (92.1%)	
Unemployed/Student	21 (6.3%)	17 (7.9%)	
Total monthly household income, n (%)			0.003
S$5000 and below	81 (24.4%)	79 (36.7%)	
S$5000-S$8000	94 (28.3%)	62 (28.8%)	
S$8000 and above	157 (47.3%)	74 (34.4%)	
Feeding practices at 6m pp, n (%)			0.755
Exclusive breastfeeding	85 (25.6%)	15 (21.4%)	
Mixed feeding	124 (37.4%)	27 (38.6%)	
Exclusive formula feeding	123 (37.1%)	28 (40.0%)	

BMI – Body Mass Index; kg – kilograms; m – months; pp - postpartum; PPWR – postpartum weight retention; SD – standard deviation; S$ - Singapore dollar.

*Other ethnic groups include Arab, Burmese, Caucasian, Eurasian, Filipino, Indonesian, Italian, Japanese, Korean, and Vietnamese.

P value was calculated from Pearson's chi-squared test or Fisher's exact test where appropriate for categorical variables and independent t test for continuous variables.

Supplementary Table S2. Association between feeding practices at 6m and PPWR based on complete dataset (n = 332)

Breastfeeding practices	β (95 % CI)				RR (95% CI)			
	PPWR at 6m		PPWR at 12m		Substantial PPWR at 6m		Substantial PPWR at 12m	
	Model 1	Model 2	Model 1	Model 2	Model 1	Model 2	Model 1	Model 2
Exclusive breastfeeding	Reference	Reference	Reference	Reference	Reference	Reference	Reference	Reference
Mixed feeding	2.24 (0.84, 3.63)	2.21 (0.81, 3.62)	1.53 (0.19, 2.88)	1.67 (0.30, 3.03)	1.68 (1.04, 2.69)	1.72 (1.06, 2.79)	1.33 (0.81, 2.19)	1.32 (0.79, 2.17)
Exclusive formula feeding	3.25 (1.86, 4.64)	3.21 (1.78, 4.64)	2.57 (1.23, 3.92)	2.62 (1.23, 4.01)	1.96 (1.23, 3.11)	1.90 (1.18, 3.07)	1.92 (1.21, 3.05)	1.80 (1.11, 2.92)

β – regression coefficient; CI – confidence interval; m – months; PPWR - postpartum weight retention; RR – risk ratio.

Model 1: crude model.

Model 2: adjusted for maternal age, pre-pregnancy body mass index, ethnicity, education level, marital status, employment status, total monthly household income, and intervention group.

Supplementary Table S3. Association between feeding practices at 6m and PPWR at 12m, stratified by pre-pregnancy BMI status

	PPWR at 12m		PPWR at 12m	
	Pre-pregnancy BMI < 23 kg/m² (n = 229)		Pre-pregnancy BMI ≥ 23 kg/m² (n = 118)	
Feeding practices at 6m pp	β (95% CI)	RR (95% CI)	β (95% CI)	RR (95% CI)
Exclusive breastfeeding	Reference	Reference	Reference	Reference
Mixed feeding	1.21 (-0.12, 2.55)	1.22 (0.67, 2.22)	3.86 (0.66, 7.05)	2.15 (0.82, 5.64)
Exclusive formula feeding	2.59 (1.20, 3.98)	1.82 (1.03, 3.20)	3.72 (0.58, 6.86)	2.23 (0.84, 5.92)

β – regression coefficient; BMI - body mass index; CI – confidence interval; pp - postpartum; PPWR - postpartum weight retention; RR – risk ratio.

Models were adjusted for maternal age, ethnicity, education level, marital status, employment status, total monthly household income, and intervention group.
